# Incidence and Trends of Leishmaniasis and Its Risk Factors in Humera, Western Tigray

**DOI:** 10.1155/2018/8463097

**Published:** 2018-09-24

**Authors:** Dawit Gebremichael Tedla, Fsahatsion Hailemariam Bariagabr, Hagos Hadgu Abreha

**Affiliations:** ^1^Aksum University Shire Campus, College of Agriculture, Veterinary Public Health, Department of Animal Science, Shire, Ethiopia; ^2^Aksum University Shire Campus, College of Agriculture, Department of Animal Science, Shire, Ethiopia

## Abstract

**Introduction:**

Leishmaniasis is a neglected vector borne disease, which constitutes a major public health concern in several tropical and subtropical countries. An estimated 4500 to 4000 new cases of visceral leishmaniasis (VL) occur per year and over 3.2 million people are at risk of infection in the country. In Humera, VL epidemics are associated with migration of workers from nonendemic highlands into the visceral leishmaniasis endemic extensive farmlands. Therefore, the objective of this study is to estimate the incidence and the risk factors of leishmaniasis in Humera, Western Tigray.

**Methods:**

A retrospective study was conducted using the hospital admission database on all patients admitted who have been suspected of having leishmaniasis infection and tested for rK39-based immune chromatographic test (ICT) at Kahsay Abera Hospital in Humera town from January 2012 to December 2017. Potential risk factors for leishmaniasis infection in human were collected from the hospital, which included categorical variables: age, sex, origin of place, clinical forms of leishmaniasis, mortality rates, and the occurrence of infections according to format of hospital.

**Results:**

A total of 26511 hospital discharged patients with diagnosis of leishmaniasis were identified, out of which 2232 (8.42%) human leishmaniasis cases were registered and of them 71 were dead from January 2012 to December 2017. Mortality rates of leishmaniasis were 18 (3.3%) in 2012, 16 (3.1%) in 2013, 15 (2.4%) in 2014, 8 (3.3%) in 2015, 9 (4.1%) in 2016, and 5 (5.4%) in 2017. Univariate analysis of the infection rate of leishmaniasis was based on the potential risk factors and found higher male infection rates than female (P <0.05) in all the study years. Origin of place was also significantly associated (P< 0.05) where labor migrants from highland to agricultural fields had higher infection rates than those who permanently lived in and around Humera. Trends in season of occurrence revealed that weeding and harvesting time (July–December) had higher incidence of leishmaniasis than dry time (January–June).

**Conclusion:**

Male labor migrants from the highlands older than 15 years of age were at the highest risks of leishmaniasis during weeding and harvest season. Therefore, awareness creation on the risks of sleeping outdoors and the impact of using of bed nets is imperative especially for labor migrants during weeding and harvesting season.

## 1. Introduction

Leishmaniasis is a serious and often fatal neglected tropical disease (NTD) which mainly affects the poorest of the poor and is associated with malnutrition, population displacement, poor housing, and a weak immune system [1, 2]. It can be caused by several species of obligate intracellular protozoan of the genus* Leishmania,* which constitutes a major public health concern in several tropical and subtropical countries [3–6]. Leishmaniasis has emerged or reemerged in many geographical areas, which increases global health and economic concerns that involved humans, domestic animals, and wild life [6]. Although human-biting sandflies occur in some other genera, the only proven vectors of human disease are the bite of phlebotomine female sand flies of the subspecies of* Phlebotomus* in the Old World and* Lutzomyia* in the New World [7–9]. There are three clinical forms of leishmaniasis: visceral (also known as kala-azar), cutaneous, and mucocutaneous. VL is the most severe form of leishmaniasis, almost always fatal if untreated [10, 11].

It is endemic in 98 countries with greater than 350 million people at risk: an estimated 700 000-1.2 million new cases, 600 000 to 1 million added new cases annually of cutaneous, 50 000 to 90 000 new cases of visceral leishmaniasis, and about 20,000 to 40,000 deaths from the disease each year [10–14]. Over 90% of the annual incidences of VL occur in six countries, namely, Bangladesh, India, Nepal, Sudan, South Sudan, Ethiopia, and Brazil [10, 14–19]. Eastern Africa is the second largest VL focus after the Indian subcontinent, which contributes to the global burden with 30,000-40,000 new cases per year [10, 14].

In Ethiopia, VL mainly occurs in the arid and semiarid areas; however, recent reports indicate spreading of the disease to areas where it was previously nonendemic [10]. The increase in leishmaniasis worldwide incidence is mainly due to the increase of several risk factors that are clearly man-made and include environmental modifications, socioeconomic status, expansion of agricultural mega projects, and new reservoir hosts [1, 20, 21].

An estimated 4,500 to 5000 new cases of VL occur per year and over 3.2 million people are at risk of infection in the country [10, 22–24]; one-third of the country's landmass is highly suitable for VL [21]. Humera town, Western Tigray, and Metema plains constitute the main VL endemic area in the country, contributing over 60% to the burden [22]. In Western Tigray, the woodland cover is in process of being replaced by extensive commercial agriculture which produces sesame as the main cash crop. The agricultural activities (weeding and harvest) attract around 200,000 seasonal labor migrants annually, mainly from the surrounding Amhara and Tigray highland areas to Humera, Western Tigray lowlands [25, 26]. VL epidemics are associated with migration and the movement of nonimmune workers from nonendemic highlands into the VL-endemic extensive farmlands [10]. Therefore, the objective of this study is to estimate the incidence and the risk factors of leishmaniasis in Humera, Western Tigray.

## 2. Methods and Materials

### 2.1. Study Area Description

**Figure 1:**
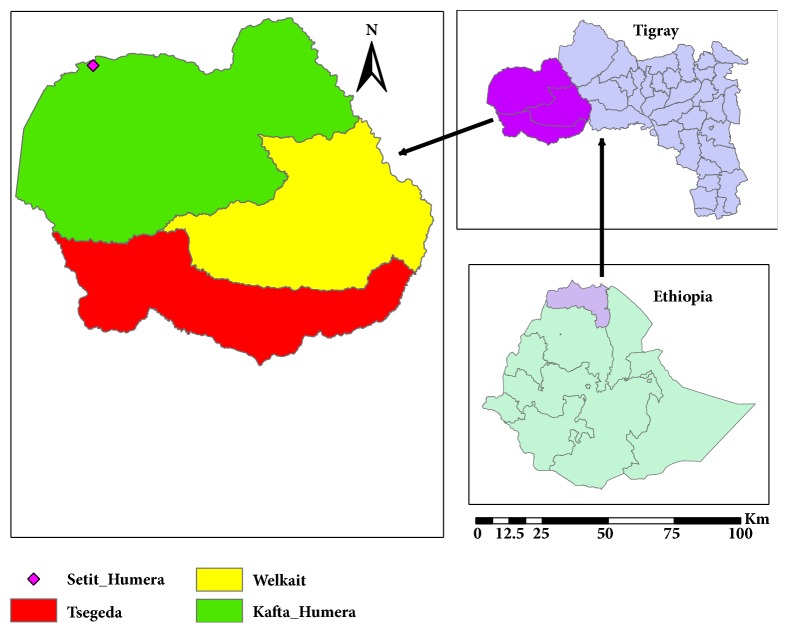
Western Tigray map showing the study area.

The study was conducted at Kahsay Abera Hospital in Humera town (Figure 1) from September to December, 2017. It is located in the western zone of the Tigray Region at longitude and latitude 14°18′N 36°37′E, with an elevation of 585 meters above sea level and the Tekeze River runs to the west. By road, it is 984 km northwest of Addis Ababa, 515 km west of Mekelle. Humera is the last important Ethiopian city south of the border with Eritrea and Sudan and is considered to be a strategically important gateway to Sudan. The overall climate throughout the year is mild and dry. The annual rainfall ranges between 400 and 600 mm, with most of the rain falling in the rainy season (June up to September). This town has a total population of 21,653, of whom 11,395 are men and 10,258 are women. The population increases dramatically during the farming season each year, when migrant workers arrive from Amhara region and Northwestern, central and eastern zone of Tigray region. Sesame, sorghum, and Arabic gum are among the most common crops. Labor migrants engaged in removing weeds from sesame seedlings, mostly after establishing themselves in the agricultural fields. But, some labor migrants return to their home at the end of August to take care of their own farm activities at home. Yet, there are some labor migrants who would come to the lowlands in September–October to harvest [27].

### 2.2. Study Design

A retrospective descriptive study was conducted using the hospital admission database, which includes all patients who were diagnosed with leishmaniasis to estimate the incidence of six-year data from Kahsay Abera Hospital's annual disease report database.

### 2.3. Data Collection Methods

The study was conducted on all patients admitted who have been suspected of having leishmaniasis infection and tested for rK39-based immune chromatographic test (ICT) at Kahsay Abera Hospital in Humera town from January 2012 to December 2017. Potential risk factors for leishmaniasis infection in human were collected from the hospital, which included categorical variables: age (0-4, 5-14, and ≥15 years), sex (male vs. female), origin of place (came from highland to agricultural fields vs. permanently living in and around Humera), clinical forms of leishmaniasis (visceral vs. cutaneous), mortality rates, and the occurrence of infections (harvesting time from July to December vs. dry time from January to June according to format of hospital).

### 2.4. Data Management and Analysis

Data were coded, checked, and uploaded into Microsoft Excel 2010 spreadsheet computer program and analyzed using STATA version 11.0 for Windows (Stata Corp., College Station, USA). Univariate and binary logistic regression performed utilizing the same program for the first set of questions included sex, age, season, and origin. 95% confidence intervals were computed and a P value* < *0.05 was considered statistically significant.

### 2.5. Ethical Approval

Ethical approval was obtained from Aksum University Shire Campus, Research and Ethical Review Committee. Consent was also sought from the hospital administration before being involved.

## 3. Results

### 3.1. Trends of Incidence of Leishmaniasis

**Figure 2:**
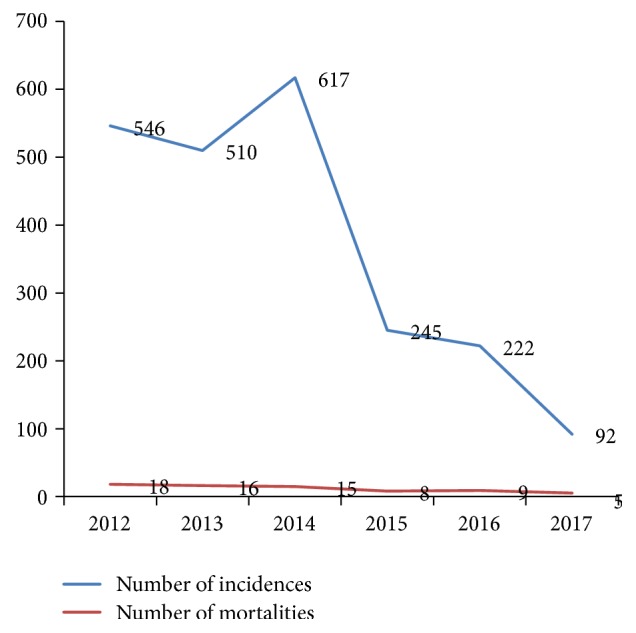
Trends of leishmaniasis in Kahsay Abera Hospital from 2012 to 2017.

A total of 26511 hospital discharged patients with diagnosis of leishmaniasis were identified, out of which 2232 (8.42%) human leishmaniasis cases were registered and from these cases, 71 were dead from January 2012 to December 2017. The results showed that leishmaniasis cases decreased across the study years with the highest recorded in 2014. Mortality rates of leishmaniasis were 18 (3.3%) in 2012, 16 (3.1%) in 2013, 15 (2.4%) in 2014, 8 (3.3%) in 2015, 9 (4.1%) in 2016, and 5 (5.4%) in 2017 (Figure 2). In the hospital leishmaniasis database, victim age was obtained and clustered in three categories: 0–4, 5 to 14, and ≥15 years. Accordingly, the highest leishmaniasis cases were recorded in the age group of ≥15 years in all the six years. Leishmaniasis cases were higher in males than females in all study years (Table 1).

Highest occurrences of the cases were those admitted during harvesting time from July to December compared to dry time from January to June in all the study years. The highest incidence of leishmaniasis was found in workers who came from highland to agricultural fields compared to those who permanently lived in and around Humera in all six years (Table 2). Regarding types of leishmaniasis, 544 (99.6%) and 2 (0.4%) in 2012, 508 (99.6%) and 2 (0.4%) in 2013, 615 (99.7%) and 2 (0.3%) in 2014, 245 (100%) in 2015, 206 (92.8%) and 16 (7.2) in 2016, and 92 (100%) in 2017 were visceral and cutaneous leishmaniasis, respectively.

### 3.2. Incidence of Leishmaniasis in relation to Risk Factors

The likelihood of infection was also significantly higher in the group greater than 15 years in all the study years. Univariate analysis of the infection rate of leishmaniasis was based on the potential risk factors and found higher male infection rates than female (P <0.05) in all the study years (Table 3). Origin of place was also significantly associated (P< 0.05) where labor migrants from highland to agricultural fields had higher infection rates than those who permanently lived in and around Humera. Trends in season of occurrence revealed that weeding and harvesting time (July-December) had higher incidence of leishmaniasis than dry time (January-June) (Table 4).

## 4. Discussions

The study provided a 6-year review of the epidemiological trends and all hospital discharged patients only to diagnose leishmaniasis in Humera. A total of 2232 human leishmaniasis cases were registered and the incidence of leishmaniasis was decreased across the study years. The reason could be mainly due to the fact that the government of Ethiopia, particularly Tigray regional state, has developed its own control strategies so as to limit the rapid spread of the disease. A national leishmaniasis task force was established in 2007 with the aim of eliminating VL and hospitals and health centers in endemic regions equipped to treat VL include Kahsay Abera Humera Hospital, Aksum Hospital, and Mekelle Hospital in Tigray regional state [28]. But, the incidence peaked in 2014 exceptionally. Sometimes, a sudden outbreak of leishmaniasis was common in this area. It may be due to the fact that the large-scale labor migrants visited this endemic area from highland.

According to available data in the Kahsay Abera Hospital, the disease was distributed in the various age groups, but occurred most frequently in the age group of greater than 15 years old (P<0.000). It might be due to the fact that matured migrant agricultural workers are highly exposed to sandfly. According to Leta* et al*. [10], all age groups are susceptible but most cases occur in groups that have regular contact with sandfly habitats. A similar result was reported where the number of patients admitted for visceral leishmaniasis was higher than that of the age group of greater than 16 years old [29, 30].

Univariate analysis revealed that being male was a risk for leishmaniasis exposure in all the study years. This gender difference might be due to difference in outdoor activity between males and females. This might be associated with activities of males in that they engage in outdoor activities such as weeding and harvesting sesame which will make them more accessible to the sandfly bite while females are more likely to remain indoors due to sociocultural factors. Similarly, sleeping under an acacia tree during the day and habitually sleeping outside at night are associated with significantly increased risk [31]. The majority of VL cases throughout the country occur in males, a pattern caused by increased exposure to the sandfly vector during agricultural work [28]. A study conducted by Oryan and Akbari [6] indicated a significant association between sex and leishmaniasis; more likely men were exposed to sandflies in fields and other open areas more than women.

Humera and its surrounding areas have significant economic input for the country because cash crops such as sesame, cotton, and sorghum are grown at a commercial scale. Thousands of male migrant workers arrive every year during the agricultural season (June–November). More than 80% of patients with VL were male migrant workers infected with* L. donovani* who sleep in the farm [29]. A similar finding was also reported in other studies [30]. This gender difference might be due to difference in outdoor activity between males and females. As indicated in another study, males are more involved in outdoor activities than females in the study area and this may have made them more susceptible to the bite of sand flies.

People who come from highlands of Tigray and Amhara regions for weeding and harvesting sesame are at higher risk of leishmaniasis infection as compared to those living permanently in and around Humera. People who came from highlands are highly exposed to sandfly due to sleeping in the farm and camp outside the house. The reason might be due to the fact that epidemics of VL are often associated with migration and the introduction of nonimmune people into areas with existing transmission cycles and most of the migrants are living outside the home. Lemma* et al*. [26] reported similar findings that people sleeping in the farm were more likely to have sero-reaction than those sleeping in the house. Following agricultural development in the area, a large number of labor migrants from the highlands were moved to the endemic areas for sesame harvesting. This led to spread of visceral leishmaniasis, which resulted in high morbidity and mortality in Humera [32]. There was a marked difference of prevalence in the farming (45.6%) and nonfarming (8.3%) communities which showed that overall skin test positivity increased with the duration of stay in the area [21].

There was statistically significant difference between infection rate and seasons in all the study years. It may be due to the fact that labor migrants are most probably exposed to leishmaniasis during June–August weeding season and staying during September–October harvest season. Epidemic outbreaks were common during weeding and harvesting times. In weeding and harvesting season, workers sleep outside the house and they could not use bed nets. Gadisa* et al*. [21] suggested that* P. orientalis* shifts from their breeding habitats to possible shelters like grass huts of labor migrants in the camp which might have increased the chance of* P. orientalis* bites or* L. donovani* infection during the weeding season in Humera.

## 5. Conclusion

Male labor migrants older than 15 years of age from highlands are the most probably exposed to leishmaniasis during June–August in the weeding season and during September–October in the harvesting season. This might be due to sleeping in the farm and camp outside the house leading to being more accessible to the sandfly bite. Therefore, awareness creation on the risks of sleeping outdoors and the impact of using of bed nets is imperative especially for male labor migrants from the highlands during weeding and harvesting season.

## Figures and Tables

**Table 1:** Trends of leishmaniasis in relation to age and sex.

Year	Demographic		No. of cases	Percent
2012	Age in years	0-4	74	13.6
		5 to 14	85	15.6
		≥15	387	70.8
		Total	546	100
	Sex	Female	74	13.6
		Male	472	86.4
		Total	546	100

2013	Age	0-4	58	11.4
		5 to 14	60	11.8
		≥15	392	76.9
		Total	510	100
	Sex	Female	63	12.4
		Male	447	87.6
		Total	510	100

2014	Age	0-4	29	4.7
		5 to 14	104	16.9
		≥15	484	78.4
		Total	617	100
	Sex	Female	39	6.3
		Male	578	93.7
		Total	617	100

2015	Age	0-4	22	9.0
		5 to 14	24	9.8
		≥15	199	81.2
		Total	245	100
	Sex	Female	27	11.0
		Male	218	89.0
		Total	245	100

2016	Age	0-4	22	9.9
		5 to 14	25	11.3
		≥15	175	78.8
		Total	222	100
	Sex	Female	38	17.1
		Male	184	82.9
		Total	222	100

2017	Age	0-4	2	2.2
		5 to 14	6	6.5
		≥15	84	91.3
		Total	92	100
	Sex	Female	3	3.3
		Male	89	96.7
		Total	92	100

**Table 2:** Trends of leishmaniasis in relation to season and origin.

Year	Season and origin		No. of cases	Percent
2012	Season	Dry time	241	44.14
		Weeding and harvesting time	305	55.86
		Total	546	100
	Origin	Lowland	248	45.4
		Highland	298	54.6
		Total	546	100

2013	Season	Dry time	207	40.59
		Weeding and harvesting time	303	59.41
		Total	510	100
	Origin	Lowland	232	45.5
		Highland	278	54.5
		Total	510	100

2014	Season	Dry time	282	45.71
		Weeding and harvesting time	335	54.29
		Total	617	100
	Origin	Lowland	201	32.6
		Highland	416	67.4
		Total	617	100

2015	Season	Dry time	97	39.6
		Weeding and harvesting time	148	60.4
		Total	245	100
	Origin	Lowland	96	39.2
		Highland	149	60.8
		Total	245	100

2016	Season	Dry time	89	40.1
		Weeding and harvesting time	133	59.9
		Total	222	100
	Origin	Lowland	99	44.6
		Highland	123	55.4
		Total	222	100

2017	Season	Dry time	30	32.61
		Weeding and harvesting time	62	67.39
		Total	92	100
	Origin	Lowland	35	38
		Highland	57	62
		Total	92	100

**Table 3:** Incidence of leishmaniasis in relation to age and sex with all diagnosed patients for leishmaniasis.

Year	Risk factors		Admitted	No. of cases	Percent	OR (95% CI)	P value
2012	Age	0-4	910	74	8.13	1.63 (1.11, 2.72)	<0.000
		5 to 14	908	85	9.36		
		≥15	1240	387	31.20		
		Total	3058	546	17.85		
	Sex	Female	1418	74	5.22	1.7(1.42-2.35)	<0.000
		Male	1640	472	28.78		
		Total	3058	546	17.85		

2013	Age	0-4	1016	58	5.71	1.8 (1.45, 2.80)	<0.000
		5 to 14	1009	60	5.95		
		≥15	1577	392	24.86		
		Total	3602	510	14.16		
	Sex	Female	1685	63	3.74	1.9( 1.1, 3.6)	<0.000
		Male	1917	447	23.32		
		Total	3602	510	14.16		

2014	Age	0-4	1103	29	2.63	1.93 (1.3, 2.96)	<0.000
		5 to 14	1352	104	7.69		
		≥15	1821	484	26.58		
		Total	4276	617	14.42		
	Sex	Female	1230	39	3.17	1.81(1.00, 3.45)	<0.000
		Male	3046	578	17.98		
		Total	4276	617	14.42		

2015	Age	0-4	1600	22	1.38	1.67(1.01, 2.02)	<0.000
		5 to 14	1680	24	1.43		
		≥15	1707	199	11.66		
		Total	4987	245	4.91		
	Sex	Female	2665	27	1.01	1.89(1.2,2.80)	<0.000
		Male	2322	218	9.39		
		Total	4987	245	4.91		

2016	Age	0-4	1494	22	1.47	1.91 (1.08, 2.72)	<0.000
		5 to 14	1582	25	1.57		
		≥15	1706	175	10.26		
		Total	4782	222	4.64		
	Sex	Female	2393	38	1.60	1.5(1.0. 2.52)	<0.000
		Male	2389	184	7.70		
		Total	4782	222	4.64		

2017	Age	0-4	1706	2	0.11	1.6 (1.1, 1.50)	<0.03
		5 to 14	1838	6	0.33		
		≥15	2262	84	3.71		
		Total	5806	92	1.60		
	Sex	Female	2710	3	0.11	1.4(0.98, 1.27)	<0.04
		Male	3096	89	2.87		
		Total	5806	92	1.60		

**Table 4:** Incidence of leishmaniasis in relation to season and origin with all diagnosed patients for leishmaniasis.

Year	Risk factors		Admitted	No. of cases	Percent	OR (95% CI)	P value
2012	Season	Dry time	1650	241	14.60	1.54(1.4, 2.52)	<0.01
		Weeding and harvesting time	1408	305	21.66		
		Total	3058	546	17.85		
	Origin	Lowland	2001	248	13.39	1.8(0.94, 2.92)	<0.000
		Highland	1057	298	28.19		
		Total	3058	546	17.85		

2013	Season	Dry time	1837	207	11.27	1.4(0.85,1.99)	<0.000
		Weeding and harvesting time	1765	303	17.17		
		Total	3602	510	14.16		
	Origin	Lowland	2423	232	9.57	1.9 (0.84, 2.92)	<0.000
		Highland	1179	278	23.58		
		Total	3602	510	14.16		

2014	Season	Dry time	2450	282	11.51	1.5(1.22, 1.97)	<0.003
		Weeding and harvesting time	1826	335	18.35		
		Total	4276	617	14.43		
	Origin	Lowland	2651	201	7.58	1.9 (1.40, 3.01)	<0.000
		Highland	1625	416	25.60		
		Total	4276	617	14.43		

2015	Season	Dry time	2870	97	3.38	1.45(1.11, 1.83)	<0.02
		Weeding and harvesting time	2117	148	6.99		
		Total	4987	245	4.91		
	Origin	Lowland	2985	96	3.22	1.6(1.7, 2.23)	<0.000
		Highland	2002	149	7.44		
		Total	4987	245	4.91		

2016	Season	Dry time	2575	89	3.46	1.6(1.6, 2.02)	<0.001
		Weeding and harvesting time	2207	133	6.03		
		Total	4782	222	4.64		
	Origin	Lowland	2866	99	3.45	1.8 (1.6, 2.18)	<0.000
		Highland	1916	123	6.42		
		Total	4782	222	4.64		

2017	Season	Dry time	2930	30	1.02	1.3(0.75, 1.6)	<0.04
		Weeding and harvesting time	2876	62	2.16		
		Total	5806	92	1.85		
	Origin	Lowland	3501	35	1.00	1.5(1.1, 1.9)	<0.001
		Highland	2305	57	2.47		
		Total	5806	92	1.85		

## Data Availability

All data generated or analyzed during this study are included in this published article. The datasets used and analyzed during the current study are available from the corresponding author on reasonable request.

## Abbreviations

CL: Cutaneous leishmaniasis
VL: Visceral leishmaniasis.
